# The Incidence of Gastrocnemius Tightness Among Clinic Staff in a District Hospital’s Clinic in the State of Kedah, Malaysia

**DOI:** 10.7759/cureus.40714

**Published:** 2023-06-21

**Authors:** Kuharajan Ramalingam, Divaagar Perumal, Harswini Balan, Juzaily F Leong, Tharumaraja Thiruselvam

**Affiliations:** 1 Orthopedics and Traumatology, Hospital Kulim/Kementerian Kesihatan Malaysia, Kulim, MYS; 2 Orthopedic and Traumatology, Hospital Canselor Tuanku Muhriz, Kuala Lumpur, MYS

**Keywords:** metatarsalgia, plantar fascitis, occupational health, foot and ankle, tendo-achillis, gastrocnemius muscle tightness

## Abstract

Aim

Musculus Gastrocnemius Tightness (MGT) has been linked with common foot and ankle pathologies. These symptoms sometimes are not severe enough for the patient to seek treatment. This study aims to determine the incidence rate of MGT among our clinical personnel and if there is any association between foot and ankle symptoms with MGT.

Materials and methods

This observational cross-sectional study involves clinical personnel from our Specialist Clinics at Hospital Kulim, Malaysia. We interviewed and assessed 85 volunteers of which, we measured the passive ankle dorsiflexion of the volunteers (the Silfverskiöld) test, to diagnose MGT. We then used the Manchester Oxford Foot Questionnaire (MOxFQ) is used to determine the functional outcome of our volunteers.

Results

Out of a total of 85 volunteers assessed, 12 (14%) volunteers were found to have gastrocnemius tightness. Among this cohort, 11 were symptomatic. Out of the 73 who did not have MGT, there were three symptomatic volunteers. There was a significant association between volunteers with foot and ankle symptoms with gastrocnemius tightness, compared to those without. There was a significant difference in the relationship between the MOxFQ scores in all components (walking, pain, and social) when comparing those with and those without MGT.

Conclusion

We conclude that there is a significant association between foot and ankle symptoms and MGT in our clinic sample population. However, these symptoms were not severe enough for these symptomatic volunteers to seek treatment. We should consider screening symptomatic staff and implementing stretching protocols.

## Introduction

Musculus gastrocnemius tightness (MGT) is defined by Baumbach as an increase in passive ankle dorsiflexion (ADF) in a flexed knee when compared to an extended knee [1,2]. It is diagnosed by a clinical examination called the Silfverskiöld test [3]. In this test, the maximum passive ADF is measured in both the extended knee (0 degree) and the knee flexed to 90 degrees. A difference of more than 13 degrees is adequate to diagnose MGT.

It is now widely known that MGT has been attributed to many foot and ankle problems. Symptoms include pain in the hindfoot, midfoot, and forefoot. Some patients may even complain of numbness, dull aches, or disturbances, with or without movement. With the increase in sedentary lifestyle, there is a possibility that a decrease in physical activity has contributed to the tightness of the gastrocnemius muscle, which, in turn, manifests as the symptoms mentioned above. While these symptoms are not exactly debilitating or cumbersome enough to cause the patient to seek medical treatment, if left unchecked, they may lead to the worsening of symptoms eventually. In our study, we aimed to determine the incidence rate of MGT among our clinic personnel and identify any association between foot and ankle symptoms and MGT.

## Materials and methods

This was an observational cross-sectional study looking into the staff of the Specialist Clinics of Hospital Kulim, Malaysia. We excluded volunteers with medical conditions that cause debilitating symptoms, such as cerebral vascular accidents, diabetes, neuromuscular diseases, and trauma (e.g., fractures of the pelvis and lower limb). Pregnant volunteers were excluded as well. We also excluded the volunteers who had previously sought treatment for their foot and ankle symptoms.

Volunteers will have had their Silfverskiöld test conducted by the primary investigator (RK). A Silfverskiöld angle of 13 degrees or more results in a positive Silfverskiöld/MGT. The lateral malleolus and the fifth ray (metatarsal) were marked with a marking pen. The test was done with the volunteer in a supine position, with the foot in a neutral position, and a commercially available digital weight scale (LazChoice GoTravel Electronic Digital Scale) was used to apply a 2-kg load on the forefoot (under the second metatarsal bone head). A commercially available inclinometer (LomVum Digital Inclinometer) was then used to measure the amount of ADF achieved in both flexed and extended knees.

The primary outcome focused on the association between MGT and foot and ankle symptoms. The cohort was screened and grouped into two groups (MGT and non-MGT) according to their Silfverskiöld test results, and further categorized according to the symptoms of each group. We took 13 degrees as a cut-off to diagnose MGT, as per Malhotra et al. Manchester Oxford Foot Questionnaire (MOxFQ) is a patient-reported outcome used for patients with foot and ankle symptoms. This questionnaire assesses how foot problems impair health-related quality of life before and after surgery.

Ethical approval was obtained from Medical Research and Ethics Committee, along with other relevant approvals prior to the start of any study-related activities. National Medical Research Register (NMRR) approval was obtained prior to data collection (NMRR ID-22-01753-EH6 (IIR) -22-01753-EH6). Data were analyzed with commercially available software, SPSS version 22 for Windows. Characteristics of the volunteers will be descriptively analyzed and summarized using frequency (N) and/percentage (%) for categorical variables, mean (μ) and standard deviation (SD), or median and interquartile range (IQR) for continuous variables. Quantitative analysis, such as a t-test and Chi-squared test, will be performed to evaluate the association of MGT with characteristics of volunteers, with mean (± SD), and mean (IQR) presented.

## Results

A total of 85 volunteers were involved in the study. The analyzed cohort consisted of 41 (48.24%) female and 44 (51.76%) male staff. The mean age of the cohort is 38.1 years old, ranging from 32 to 55 years old. The analyzed group presented a mean weight of 70.7 kg, ranging from 43 kg to 130 kg; a mean height of 164.4 cm, ranging from 150 cm to 185 cm; and a mean body mass index (BMI) of 26.1 kg/m^2^, ranging from 16.4 kg/m^2^ to 43.4 kg/m^2^. The incidence rate of MGT was found higher in the right leg (proportion: 14.12, 95% CI: 6.72, 21.52) than in the left leg (proportion: 7.06, 95% CI: 1.61, 12.50); however, the difference was not statistically significant (p-value (p) = 0.1348) (Table 1).

**Table 1:** Descriptive statistics of 85 volunteers included in the analysis and incidence rate of MGT according to right and left legs. SD=standard deviation, N=frequency, %=percentage, 95% CI=95% confidence interval

Variables	Group	N	Median	Mean (±SD)	Min, Max	Proportion (95% CI)
Age, years		85	38	38.1 (±7.3)	32, 55	
Gender	Female	41				48.23
	Male	44				51.76
Weight, kg		85	70	70.7 (±14.8)	43, 130	
Height, cm		85	164	164.4 (±8.1)	150, 185	
Body Mass Index, kg/m^2^		85	25.7	26.1 (±4.8)	16.4, 43.4	
Right leg						
Silfverskiöld			7.5	8.1 (±4.4)	1, 21.3	
Gastrocnemius muscle tightness (MGT)	Yes	12				14.12 (6.72, 21.52)
Symptoms	Yes	14				16.47 (8.59, 24.36)
Walking	(>0)	11				12.94 (5.81, 20.08)
Pain	(>0)	13				15.29 (7.64, 22.95)
Social	(>0)	10				11.76 (4.92, 18.61)
Index Score			0	3.2 (±9.6)	0, 54.7	
Left leg						
Silfverskiöld			7.4	7.4 (±3.6)	0.7, 19.1	
Gastrocnemius tightness	Yes	6				7.06 (1.61, 12.50)
Symptoms	Yes	10				11.76 (4.92, 18.61)
Walking	(>0)	8				9.41 (3.20, 15.62)
Pain	(>0)	10				11.76 (4.92, 18.61)
Social	(>0)	9				10.59 (4.05, 17.13)
Index Score			0	2.4 (±8.0)	0, 51.5	

The most important result obtained was a noticeably higher incidence rate of MGT, which was also significantly associated with more symptoms and pain (p<0.0001). There was a significant difference in the relationship between the MOxFQ scores in all components (walking, pain, and social) when comparing those with and those without MGT (Table 2).

**Table 2:** Symptoms and pain associated with the presence of MGT.

Variables	Group	With MGT (N=12)	No MGT (N=73)	Proportion difference	P-value
Symptoms	Yes	11 (91.7)	3 (4.1)	87.6	<0.0001
Walking	(>0)	9 (75.0)	3 (4.1)	70.9	<0.0001
Pain	(>0)	11 (91.7)	3 (4.1)	87.6	<0.0001
Social	(>0)	9 (75.0)	3 (4.1)	70.9	<0.0001

In general, males presented a higher incidence rate of MGT in the right leg (15.9% for males versus 12.2% for females, p=0.6231), while females presented a higher incidence rate of MGT in the left leg (9.8% for females versus 4.5% for males, p=0.4227). However, due to the small sample size, these findings were not statistically significant (p>0.05) (Table 3).

**Table 3:** Incidence rate of MGT and symptoms according to gender.

Variables	Group	Female (N=41)	Male (N=44)	Proportion difference	P-value
Right leg					
Gastrocnemius tightness	Yes	5 (12.2)	7 (15.9)	– 3.7	0.623
Symptoms	Yes	6 (14.6)	8(18.2)	– 3.6	0.660
Walking	(>0)	5 (12.2)	6 (13.6)	– 1.4	0.843
Pain	(>0)	6 (14.6)	7 (15.9)	– 1.3	0.870
Social	(>0)	4 (9.8)	6 (13.6)	– 3.8	0.740
Left leg					
Gastrocnemius tightness	Yes	4 (9.8)	2 (4.5)	5.3	0.422
Symptoms	Yes	5 (12.2)	5 (11.4)	0.8	0.905
Walking	(>0)	4 (9.8)	4 (9.1)	0.7	0.916
Pain	(>0)	5 (12.2)	5 (11.4)	0.8	0.905
Social	(>0)	4 (9.8)	5 (11.4)	– 1.6	0.810

## Discussion

Clinic staff with symptomatic foot and ankle pain, as well as increased MOxFQ scores, were found to be associated with isolated GMT. Possible reasons for this would be extended duration of standing and walking during clinic hours as well as the type of shoes worn by the staff. These symptoms were not yet severe enough (possibly) for the individuals to seek any treatment. There was a significant increase in all segments of the MOxFQ in the MGT group compared to the normal group. Patients presenting with unilateral foot pathologies, such as plantar ulcerations, metatarsalgia, plantar fasciitis, etc., should be examined for impaired ADF and MGT [2]. Although there is some consideration of reduced ADF with knee extension as age advances [4], our sample population had a mean age of 38+/-7 years.

GMT predisposes individuals to multiple musculoskeletal conditions, as well as various foot pathologies. There is also a link between patients with symptomatic forefoot pathologies and a higher body mass index, as well as lower activity levels [5]. When compared to a control population, DiGiovanni found that patients with midfoot or forefoot symptoms had an almost three times higher prevalence of isolated MGT [6]. Lau quantified the relationship between MGT and the severity of heel pain in plantar fasciitis [7]. Barouk found a link between hallux valgus as a common consequence of MGT, attributing the links between tendo-achillis, calcaneus, and plantar aponeurosis to the sesamoids and the hallux [8]. Huerta summarized that MGT increases Achilles tndon tension during weight bearing, leading to an increase in forefoot pressure with an anterior displacement of the center of pressure [9]. Cazeau concludes that MGT has a biomechanical effect on the lower limb joints and the forefoot, with the risk of forefoot pain when the associated muscles are maximally stretched [10]. Nakale et al., in a study of 223 patients, found a strong association between plantar fasciitis, osteoarthritis, and hallux valgus, among others, with isolated MGT [11]. Using muscle reaction time, Lee et al. found patients with plantar fasciitis had weakness not only in the gastrocnemius muscle but also in the hamstrings [12].

Treatment for MGT begins with conservative management. Stretching exercises have been recommended throughout the literature and have yielded good results. It is a cost-effective approach and can be performed by patients on their own. Bernand and Cazeu believe that stretching exercises should be the first line of treatment [7,10]. If this fails, there is a Grade B recommendation for gastrocnemius recession surgery for overload symptoms (isolated pain) [13].

As mentioned, the purpose of this study is to identify a cohort of patients who have foot and ankle symptoms that were not severe enough for them to seek medical treatment. While our sample size is small and a larger wider sample size is warranted, there is an association between MGT and foot/ankle complaints.

This study has some limitations. Although all the volunteers were adults and employed in the clinic, no analysis was done into their level of physical activity. The use of BMI in our study may also bring about some debate, as it does not take into account the fat to muscle ratio. Although this is outside the scope of our study, the usage of abdominal girth may be considered against the use of BMI.

## Conclusions

In our clinic sample population, there was a 14% incidence of MGT. We found an association between foot and ankle symptoms and MGT, which are not severe enough for patients to seek treatment. The stretching exercises, in addition to being cost-effective and requiring no special equipment, could potentially reduce patient symptoms and address problems before they worsen.

We propose a larger-scale study involving our medical teams, including those in intensive care/operating theatres. This study will compare the cohort prospectively, using a standardized gastrocnemius muscle stretching exercise protocol. The results from these studies can inform the implementation of stretching exercises in community outreach programs, as well as serve as a screening tool in community clinics and pre-specialist clinic protocols. This will enable primary care physicians to educate patients on gastrocnemius muscle stretching.

## References

[1] Baumbach SF, Braunstein M, Regauer M, Böcker W, Polzer H (2016). Diagnosis of musculus gastrocnemius tightness - key factors for the clinical examination. J Vis Exp.

[2] Baumbach SF, Braunstein M, Seeliger F, Borgmann L, Böcker W, Polzer H (2016). Ankle dorsiflexion: what is normal? Development of a decision pathway for diagnosing impaired ankle dorsiflexion and M. gastrocnemius tightness. Arch Orthop Trauma Surg.

[3] Barouk P, Barouk LS (2014). Clinical diagnosis of gastrocnemius tightness. Foot Ankle Clin.

[4] Chan O, Malhotra K, Buraimoh O, Cullen N, Welck M, Goldberg A, Singh D (2019). Gastrocnemius tightness: a population based observational study. Foot Ankle Surg.

[5] Malhotra K, Chan O, Cullen S, Welck M, Goldberg AJ, Cullen N, Singh D (2018). Prevalence of isolated gastrocnemius tightness in patients with foot and ankle pathology: a population-based study. Bone Joint J.

[6] DiGiovanni CW, Kuo R, Tejwani N, Price R, Hansen ST Jr, Cziernecki J, Sangeorzan BJ (2002). Isolated gastrocnemius tightness. J Bone Joint Surg Am.

[7] Pearce CJ, Seow D, Lau BP (2021). Correlation between gastrocnemius tightness and heel pain severity in plantar fasciitis. Foot Ankle Int.

[8] Barouk LS (2014). The effect of gastrocnemius tightness on the pathogenesis of juvenile hallux valgus: a preliminary study. Foot Ankle Clin.

[9] Pascual Huerta J (2014). The effect of the gastrocnemius on the plantar fascia. Foot Ankle Clin.

[10] Cazeau C, Stiglitz Y (2014). Effects of gastrocnemius tightness on forefoot during gait. Foot Ankle Clin.

[11] Nakale NT, Strydom A, Saragas NP, Ferrao PN (2018). Association between plantar fasciitis and isolated gastrocnemius tightness. Foot Ankle Int.

[12] Lee JH, Jung HW, Jang WY (2020). A prospective study of the muscle strength and reaction time of the quadriceps, hamstring, and gastrocnemius muscles in patients with plantar fasciitis. BMC Musculoskelet Disord.

[13] Cychosz CC, Phisitkul P, Belatti DA, Glazebrook MA, DiGiovanni CW (2015). Gastrocnemius recession for foot and ankle conditions in adults: evidence-based recommendations. Foot Ankle Surg.

